# Investigation and Optimisation of the Rheological Properties of Magnesium Potassium Phosphate Cement with Response Surface Methodology

**DOI:** 10.3390/ma15196815

**Published:** 2022-09-30

**Authors:** Yanfei Yue, Jun Ren, Kai Yang, Danqian Wang, Jueshi Qian, Yun Bai

**Affiliations:** 1 College of Materials Science and Engineering, Chongqing University, 174 Shazheng Street, Shapingba, Chongqing 400044, China; 2 School of Architecture and Planning, Yunnan University, Kunming 650051, China; 3 Department of Civil, Environmental and Geomatic Engineering, University College London, Gower Street, London WC1E 6BT, UK; 4 School of Civil Engineering, University of Leeds, Woodhouse Lane, Leeds LS2 9JT, UK

**Keywords:** magnesium potassium phosphate cement, mini slump, plastic viscosity, response surface methodology, rheological properties, yield stress

## Abstract

Magnesium phosphate cement (MPC) is a promising alternative cement. However, the rheological property of this new binder is still to be explored. In this study, Response Surface Methodology (RSM) was adopted with Central Composite Design (CCD) to establish mathematical models describing the rheological characteristics of MPC in terms of initial mini slump (Y_1_), mini-slump loss (Y_2_), yield stress (Y_3_) and plastic viscosity (Y_4_), as a function of three independent variables, namely, water-to-solid ratio (W/S ratio, X_1_), MgO to MKP ratio (M/P ratio, X_2_) and borax dosage (X_3_). The results show that the M/P ratio and borax dosage could significantly affect the yield stress and mini-slump loss of MPC, while the W/S ratio was the significant coefficient influencing plastic viscosity and initial mini slump. The numerical optimised values of X_1_, X_2_ and X_3_ were 0.280, 7.528 and 0.170, respectively, and an MPC paste with desirable rheological characteristics (Y_1_ 161.858 mm, Y_2_ 11.282, Y_3_ 0.680 Pa, Y_4_ 0.263 Pa·s) with the highest desirability of 0.867 can be obtained.

## 1. Introduction

Magnesium phosphate cement (MPC), also known as chemically bonded phosphate ceramic, is an alternative clinker-free binder which has attracted increasing attention worldwide [1,2,3,4,5]. Compared to the traditional cement [6], MPC possesses many superior properties, such as super-fast setting, high early strength, strong bonding, low drying shrinkage and better durability [1,2,3,4,5]. These properties make MPC remarkably popular in the fast repair, strengthening and rehabilitation of concrete infrastructures such as pavements, highways and airport runways [7,8]. In recent decades, MPC has also demonstrated huge potential for stabilising/solidifying heavy metals, immobilising nuclear wastes, as well as 3D-printing [9,10]. As a promising alternative cement, the hydration and hardening mechanisms of MPC have been extensively studied in the past [11,12,13,14]. In general, the hydration of MPC is considered to be a through-solution acid-base exothermic reaction between dead-burnt magnesia (MgO) and an acid phosphate source in the presence of water to form magnesium phosphate hydrates [11,12,13,14]. Although monoammonium phosphate (NH_4_H_2_PO_4_, MAP) has widely been employed as the source of phosphate in earlier studies in the literature, monopotassium phosphate (KH_2_PO_4_, MKP) has increasingly been employed in recent years owing to its lower solubility than ammonium phosphates, which can slow down the violent reaction and reduce the heat released. Additionally, using MKP also has the advantage of avoiding the emission of unpleasant ammonium gas [14]. The main hydration product of the MgO–KH_2_PO_4_ system is MgKPO_4_·6H_2_O (k-struvite), and its composition and structure are reported to be analogous to those of NH_4_MgPO_4_·6H_2_O (struvite) formed in the MgO–NH_4_H_2_PO_4_ system [5,14,15]. Different from the poorly ordered calcium silicate hydrate (C-S-H) gel formed in PC, the magnesium phosphate hydrates formed in MPC are mainly crystalline phases, and thus, its volume stability is believed to be better than PC-based materials [16,17].

Although the fundamentals regarding MPC—such as its hydration mechanisms, the properties of the hydrates, factors affecting the properties, as well as its potential applications—have been extensively investigated [13,14,18,19], its rheological properties are rarely reported and largely unknown. Whilst the rheological properties of conventional PC-based cementitious materials have been well established and commonly described using a Bingham model defined by yield stress (τ) and plastic viscosity (μ) [20], studying the rheological properties of MPC could be rather challenging. This could partly be due to its super-fast setting, making it extremely difficult to characterise its fresh properties, such as rheology. Nonetheless, to efficiently utilise MPC-based materials in industrial applications, their rheological behaviour needs to be understood and optimised. This will also significantly benefit the transporting, placing, and finishing processes in engineering practice. Apart from its super-fast setting, the two main constituents of MPC could also cause complex issues to the fresh properties of MPC. This is because, even though the water-to-solid (W/S) ratio is widely established and expected to affect the rheology of MPC system, the MgO to MKP (M/P) ratio can also cause uncertainties to the properties of MPC. For example, it has been shown that the compressive strength of MPC is not a simple monotonic function of M/P. Instead, at a given W/S, there is always an optimal M/P for the highest compressive strength [8], which is different from what has been widely anticipated, i.e., the higher the M/P, the higher the compressive strength. In terms of rheology, whether such kinds of complex also exist is still unknown in the literature. Furthermore, except for the MgO and phosphate salt, a ‘third’ part, termed as retarder, is usually incorporated in the manufacture of MPC to modulate the setting time. This retarding agent, normally boric acid (H_3_BO_3_) or borate (Na_2_B_4_O_7_·5H_2_O), could introduce further uncertainty and complexity to the fresh properties of MPC [12,21]. Due to the above issues, performance-based design approaches have been widely adopted by different researchers to obtain the desired strength properties. Although this trial-error based method has also been extensively used in the laboratory to formulate the MPC with targeted fresh properties, it is not only time-consuming, but also resource-intensive. Moreover, even though this performance-based approach could allow a mix with good performance to be identified, it cannot ensure a mix with the optimal performance being always achieved. Therefore, Response Surface Methodology (RSM) is attempted as a potential mathematical tool in this paper to resolve the above complexes in order to optimise the rheological properties of MPC based on systematic numerical analyses.

Response Surface Methodology (RSM) is a mathematical and statistical approach using empirical modelling to explore the relationships between independent variables (explanatory factors) and the responses of interests, as well as the interactions between the independent variables [22]. Compared to the standard experimental methods, RSM makes a research strategy systematic and efficient to correlate the interactive effects of numerous parameters simultaneously with the help of statistical procedures. The Central Composite Design (CCD) is the most used surface response statistical design method, which runs much fewer experiments than a full factorial design but provides almost the same information [23]. Hence, the RSM jointly used with the statistic design method (e.g., CCD) is recognised as a significant approach in the design and optimisation process, which has also recently been effectively employed to investigate the interactive effects of independent variables in many areas [24,25,26]. In the field of cement and concrete research, RSM demonstrated great potential for experimental optimisation, particularly in optimising the rheological parameters, hydration and mechanical properties of cementitious materials [27,28,29,30]. Recently, Hou et al. [31] presented their research on optimising the setting time and early-age compressive strength of MPC cement with RSM methodology for rapid repair applications. Using water-to-binder ratio, magnesia-to-phosphate ratio and borax content as the variables, and the setting time and early-age compressive strength as the responses in RSM, a statistical model was successfully developed by Hou et al. [31], showing a good potential of applying RSM for the design and optimisation of MPC mix. However, the rheological parameters (e.g., mini slump, mini-slump loss, yield stress and plastic viscosity) of MPC were not investigated in their study.

Building upon the aforementioned information, it is anticipated that RSM-CCD methodology could be used to explore the rheological property of this new MPC materials. Hence, in this study, the RSM methodology was adopted to investigate the rheological behaviour of MPC material and also to optimise the mix proportion in terms of water/solid ratio (W/S), MgO/MKP ratio (M/P) and borax dosage, with initial mini slump, mini-slump loss, yield stress (τ, Pa) and plastic viscosity (μ, Pa·s) considered as responses. The CCD design was, therefore, conducted to develop a three variables (factors) (n = 3) experiment matrix with 20 runs in total. In addition, the analysis of variance (ANOVA) was conducted to assess the significance and adequacy of the regression models attained. Finally, to supplement the mathematical analyses, the early-age (i.e., 1 d and 7 d) compressive strength of the MPC pastes was investigated to verify its application in real engineering practices.

## 2. Materials and Methods

### 2.1. Materials

Magnesium oxide (MgO) provided by Richard Baker Harrison Ltd., Manchester, UK, was a Dead Burned Magnesite (DBM) calcined at about 1750 °C with a purity of 90% and a mesh size of 200 μm. Monopotassium phosphate (KH_2_PO_4_, MKP) was a food-grade MKP with a specified purity >99%, from Prayon UK. Sodium tetraborate decahydrate (borax, Na_2_B_4_O_7_·10H_2_O), supplied by Sigma-Aldrich US, was employed as the retarder. It was ACS reagent grade with assay ≥99.5% and a pH of 9.15–9.20.

### 2.2. Design and Sample Preparation

**Figure 1:**
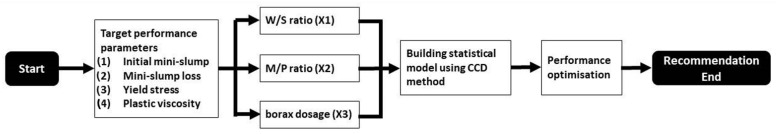
Illustration of overall experimental design for this study.

The work process contains three steps: (1) selecting targeted performance parameters and key influencing factors; (2) building and fitting the statistical models for each performance parameter; (3) optimising the mix proportion to achieve the aimed performance. The overall framework for this study is illustrated in Figure 1.

**Figure 2:**
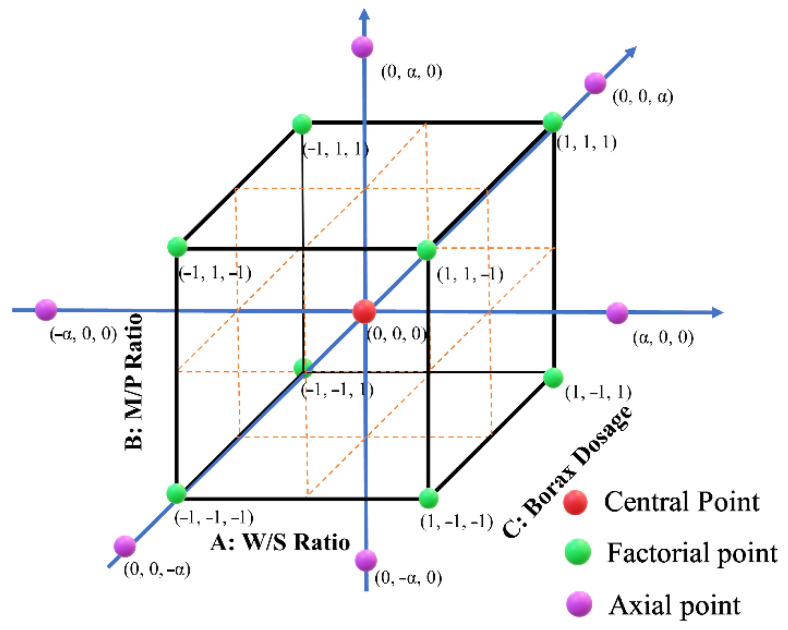
Schematic of the design points with coded values.

A typical CCD design involves 2^n^ factorial points, 2^n^ axial points, and n_c_ centre points, in which *n* represents the number of factors/variables, and the n_c_ refers to the number of repetitions of the centre point. The repetition of the central point is to estimate the experimental internal error and, thus, to improve the reliability of the models. In this study, a 2^3^ CCD design with three variables (n = 3) and two extreme levels (coded as +α and −α) was applied to develop the mathematical equation and quantify the rheological properties of MPC in terms of initial mini slump, mini-slump loss, yield stress and plastic viscosity. The three variables investigated in this study were water/solid ratio (W/S) (X_1_), MgO/MKP ratio (M/P) (X_2_) and borax dosage (X_3_) (by the weight of MgO). A total of 20 runs, including eight (2^n^ = 2^3^) factorial points, six (2n = 2 × 3) axial points and six (replicates) centre points, were carried out. The CCD design and data analysis associated with the analysis of variance (ANOVA) and RSM optimisation were conducted using Design Expert 10 (Stat-Ease, Minneapolis, MN, USA) software. Table 1 below reports the actual values of the factors, and Figure 2 visually illustrates the distribution of the coded points for the designed matrix. It should be noted that the ranges/levels of the variables were determined based on the results from our previous work [32]. In total, 20 MPC pastes were designed by the CCD method and the details of the coded value of mixing portions are given in Table 2.

All 20 MPC pastes, as shown in Table 2, were fabricated in a high-shear mixer. The borax was first mixed with water and followed by adding MKP and MgO. The total mixing time was 3 min.

### 2.3. Test Methods

#### 2.3.1. Rheological Properties Test

Immediately after mixing, the pastes were transferred into the container (92 mm diameter) of a modified and calibrated rheometer, which uses a helical impeller to establish the relationship between shear stress and shear rate. The results were recorded by the software and the yield stress and plastic viscosity were fitted by the Bingham model. The mini-slump test was conducted with a conical mould with a lower inner diameter of 38.1 mm, an upper inner diameter of 19 mm and a height of 57.1 mm. The mini slump was determined at 15 min and 45 min after mixing. The mini-slump loss was then calculated as the difference between the values of mini slump at 15 min and 45 min.

#### 2.3.2. Compressive Strength Test

The 25 × 25 × 25 mm^3^ PVC moulds were used to cast cubes to determine the compressive strength of the paste. Immediately after mixing, the moulds were filled with the paste and a steel scraper was used to finish the specimen surface. The moulds were covered with water-saturated hessian and stored in a constant temperature room at 20 ± 1 °C for 24 h. The specimens were then demoulded, and each cube was wrapped with a water-saturated hessian before being sealed in an air-tight plastic bag. The specimens were again stored in the same constant-temperature room until being tested.

### 2.4. Data Analysis

The data were processed by Design Expert software. The analysis of variance (ANOVA) with Fisher’s F-test was conducted to check the significance and adequacy of the models established. The desirability function (DF) approach was used to establish the optimum criteria based on multi-variables. The general procedure of this approach is to convert each response $Y_{k}$ into an individual desirability function as $\;d_{k}=f\left(Y_{k}\right)\;\left(0\;\leq \;d_{k}\leq 1\right)$. If the response ($Y_{k}$) meets the target of the optimisation goal, $\;d_{k\;}$ is then valued as 1; while the response is beyond the acceptable limit, then $d_{k}$ is 0.

## 3. Results and Discussion

### 3.1. Model Adequacy Analysis

The experimental data in terms of the four responses (i.e., Y_1_, Y_2_, Y_3_ and Y_4_) obtained from all 20 mixes are presented in Table 3 and analysed by Design Expert 10. The best-fitting surface response model for describing the mini slump (Y_1_) is generated, which is suggested to be a linear relation, whilst the models of the other three responses, i.e., mini-slump loss (Y_2_), yield stress (Y_3_), and plastic viscosity (Y_4_), are two-factor interactions (2FI). The regression equations in the coded value attained correlating to the responses and the three independent variables are shown in Equations (1)–(4) for initial mini slump, mini-slump loss, yield stress and plastic viscosity, respectively. The analysis of variance (ANOVA) with Fisher’s F-test was then conducted to assess the significance and adequacy of these models, which are reported and discussed in separate sections (Section 3.1.1, Section 3.1.2, Section 3.1.3, Section 3.1.4 and Section 3.1.5) below. Since the repeatability of the responses at the central points was used for estimating the error of the models, the analysis of the results of the four responses at central points was also conducted, and the results are presented in Table 4.

Y_1_ = 153.60 + 9.48X_1_ + 0.14X_2_ − 1.24X_3_ (1)

Y_2_ = 38.75 − 9.84X_1_ + 28.68X_2_ − 13.55X_3_ + 3.87X_1_X_2_ − 5.62X_1_X_3_ − 23.88X_2_X_3_ (2)

Y_3_ = 9.61 − 9.67X_1_ + 11.60X_2_ − 10.41X_3_ − 14.25X_1_X_2_ + 14.33X_1_X_3_ − 15.23X_2_X_3_ (3)

Y_4_ = 0.73 − 0.43X_1_ − 0.04X_2_ + 0.20X_3_ + 0.24X_1_X_2_ − 0.37X_1_X_3_ + 0.52X_2_X_3_ (4)

#### 3.1.1. Analysis of Variance (ANOVA) of the Response Model of Initial Mini-Slump

The analysis of variance of the model of initial mini slump (Equation (1)) is shown in Table 5. The obtained F-value of 9.67 and p-value of 0.0007 (<0.05) implies that the regression model was highly significant at a 5% significance level, and there was only a 0.07% chance that an F-value this large could occur due to noise. The goodness of fit of the model was assessed by the coefficient of determination R^2^ and equalled to 0.6446, which indicates that about 64.46% of the variation in this system was attributed to the independent variables, and only about 35% of the variation could not be explained by the model. In addition, the adequate precision value measures the signal-to-noise ratio and a value greater than 4 is desirable. The value obtained from the current model was 10.8699, suggesting that the adequate signal was obtained, and this model can be used to navigate the design space. Moreover, the significance of each of the coefficients can be checked by the p-value, as listed in Table 5, and p < 0.05 could suggest that the term is significant. From our results, evidently, the X_1_ is the significant model term. This suggests that the factor W/S (X_1_) is the most significant term for the linear model of mini slump, which significantly affects the initial mini slump of the MPC paste.

#### 3.1.2. Model Describing Mini-Slump Loss

Similarly, the ANOVA analysis with Fisher’s F-test was conducted to check the significance and adequacy of the model of mini-slump loss (Equation (2)), which is presented in Table 6. The F-value and p-value of the 2FI model were 5.18 and 0.0063, respectively, revealing that the model was significant. Moreover, the coefficient of determination (goodness of fit) was R^2^ = 0.7051, which indicates that 70.51% of the variation in this system can be attributed to the independent variables. In addition, the value of adequate precision was 7.6423, which suggests that the model can be validly used in the examined range of experiments. Moreover, by checking the p-value of all the terms, the factors of X_2_ (0.0011) as well as X_2_X_3_ (0.0195) were significant model terms, which suggests that M/P (X_2_) significantly affects the mini-slump loss of MPC (95% confidence limit), and there are significant interactions between M/P and borax dosage (X_2_X_3_). On the other hand, the factor borax dosage (X_3_) was still within the 90% confidence limit, which also somehow showed the influence on the mini-slump loss of MPC.

#### 3.1.3. Model Describing Yield Stress

The statistical significance and adequacy of the response model of yield stress (Equation (3)) were checked, and the results are shown in Table 7. The response model was significant, as evidenced by the F-value and p-value which were 5.13 and 0.0066, respectively. Moreover, the coefficient of determination R^2^ equalled to 0.7030 (goodness of fit)*,* indicating that 70.30% of the variation in this system can be attributed to the independent variables, and only about 30% of the variation cannot be explained by the model. The lower value of R^2^ could be due to the indirect response (yield stress) from the factors. The value of adequate precision obtained from the current model was 9.6694 (greater than 4), suggesting that the adequate signal was obtained and this model is valid to navigate the design space. Moreover, by checking and comparing the p-value listed in Table 7, it is evident that all the factors and interactions, excepting W/S (X_1_), were identified as significant model terms at a 95% confidence limit. Based on the statistical analysis, the M/P (X_2_) and borax dosage (X_3_) could significantly affect the yield stress of the MPC materials, and there are significant interactions between W/S and M/P (X_1_X_2_), W/S and borax dosage (X_1_X_3_) and M/P and borax dosage (X_2_X_3_). Furthermore, the p-value of W/S (X_1_) was 0.0655, which is still acceptable within the allowance of the 90% confidence limit.

#### 3.1.4. Model Describing Plastic Viscosity

The ANOVA analysis for the model of plastic viscosity (Equation (4)) is shown in Table 8. The current model, with a p-value of 0.0979 (which is less than 0.1), could still be considered as significant at a 10% significance level. The determination coefficient R^2^ obtained was 0.5151, which suggests that this model can interpret 51.51% of the total variation. Moreover, the adequate signal was retrieved, as evidenced by the adequate precision value of 6.1537. More importantly, the X_1_ was observed to be the only significant coefficient in the current model, which implies that the W/S ratio played an important role in adjusting the viscosity of the MPC materials.

#### 3.1.5. Summary and Further Improvement

In this section, in addition to the establishment of four prediction models with the analysis factors and their interactions, an ANOVA was carried out, and the influence of the main factors was assessed along with the interaction. For all the models, the p-values were less than 0.1, indicating that these four models are significant at a 10% significance level. Furthermore, based on the analysis of each factor and their interactions, the most significant terms for each model were identified, as discussed in Section 3.2.5. However, two improvements could be further applied: (1) the results indicated strong interactions between the factors affecting mini-slump loss and yield stress, which could be further analysed; (2) the values of R^2^ of the four models were below 0.85 and the lowest value was 0.5151, indicating that there is some room to improve these models.

### 3.2. Effect of Variables on the Response of the Model

In this section, based on the statistical analysis of the four predicted models, the effects of different variables and their interactions in each predicted model are discussed.

#### 3.2.1. Effect on Initial Mini-Slump

**Figure 3:**
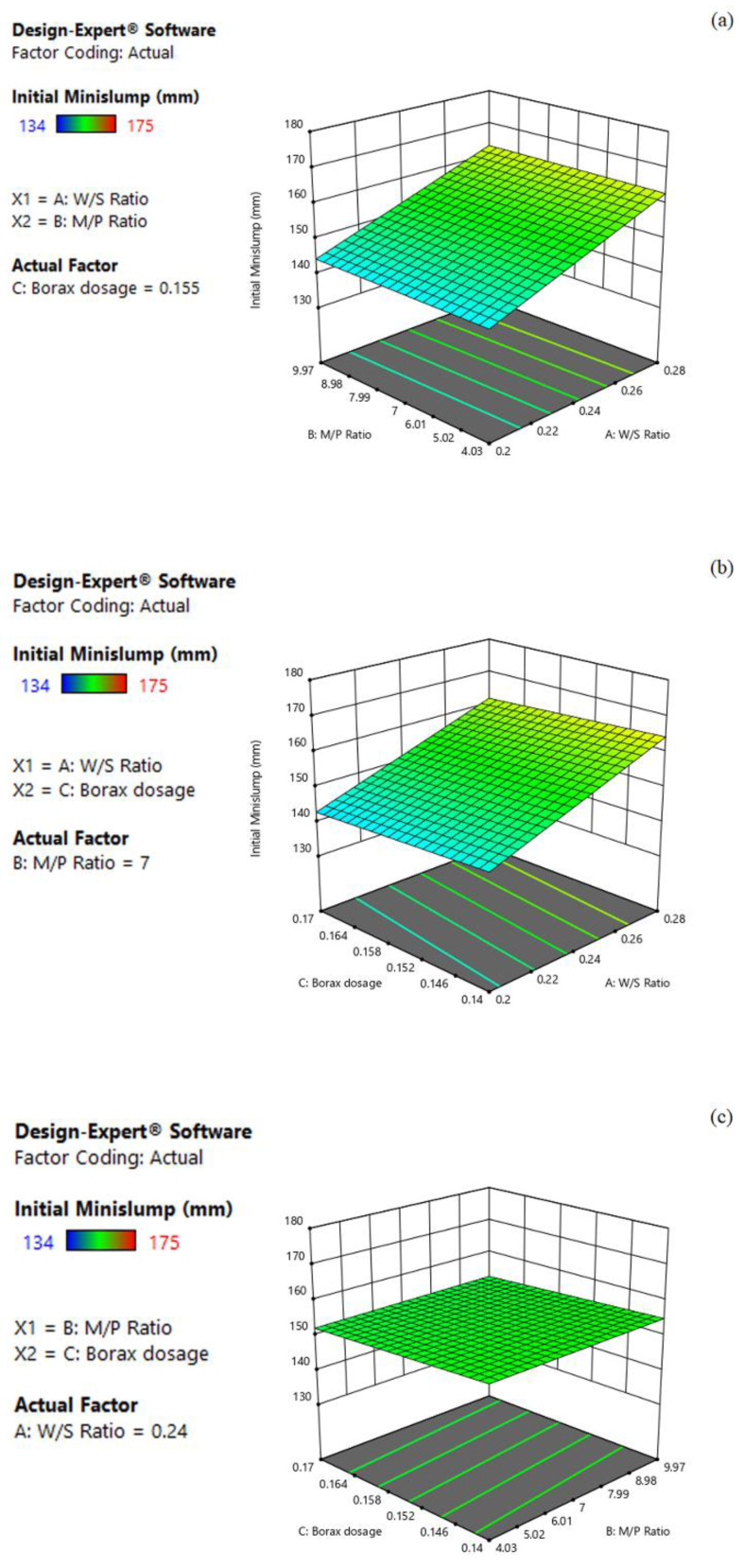
Three-dimension response surface plots of initial mini slump (Y_1_) in relation to: (**a**) W/S ratio (X_1_) and M/P ratio (X_2_); (**b**) W/S ratio (X_1_) and borax dosage (X_3_); (**c**) M/P ratio (X_2_) and borax dosage (X_3_).

The 3D response surface plots of the initial mini slump with an interactive relationship between the factors (W/S, M/P, borax dosage) are shown in Figure 3a–c. Based on Equation 1, the initial mini-slump response increased with the increase in the W/S ratio and M/P ratio, whereas it decreased with the increase in borax dosage. Moreover, the higher coefficient of the W/S, which was approximately 67.1 and 7.6 times larger than those of M/P and borax dosage, respectively, indicates that the W/S significantly affected the initial mini slump of MPC. As shown in Figure 3a, the initial mini slump increased with the increase in W/S, whereas the change of the M/P imposed little effect on it. For example, the response of the predicted mini slump was 153.60 mm when the values of W/S and M/P were set as 0.24 and 7, respectively. However, when increasing the W/S to 0.28 while maintaining M/P at 7, the predicted initial mini slump increased to 163.07 mm, while it only increased to 153.73 mm if increasing M/P to 9.97 but keeping the W/S at 0.24. On the other hand, the effects of W/S and borax dosage on the initial mini slump are presented in Figure 3b. Similarly, the initial mini slump increased as the W/S increased towards its high level. In contrast, the initial mini slump decreased along with increasing borax dosage. In addition, as illustrated in Figure 3c, the initial mini slump increased with decreasing borax dosage, while the change in M/P provided little effect on it.

#### 3.2.2. Effect on Mini-Slump Loss

**Figure 4:**
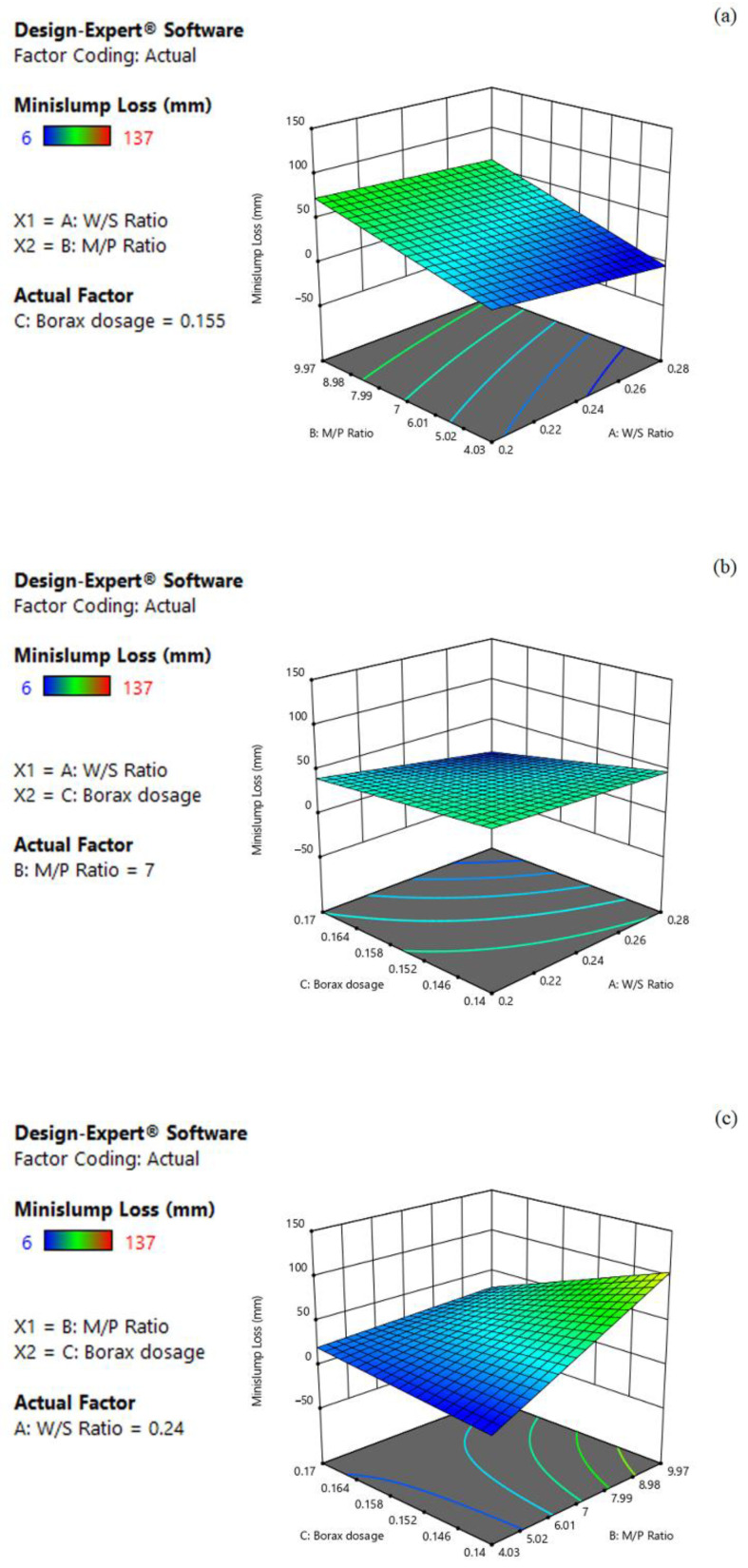
Three-dimension response surface plots of mini-slump loss (Y_2_) in relation to: (**a**) W/S ratio (X_1_) and M/P ratio (X_2_); (**b**) W/S ratio (X_1_) and borax dosage (X_3_); (**c**) M/P ratio (X_2_) and borax dosage (X_3_).

The 3D response surface plots visualizing the effect of the three factors on the mini-slump loss of MPC paste are plotted in Figure 4. Generally, the mini-slump loss response decreased with the increase in W/S and borax dosage, while it increased with the increase in M/P. Moreover, the influences in the order of decreasing magnitude are M/P, borax dosage and W/S. For example, the coefficient of M/P showed an approximately 2.11 times greater effect on the mini-slump loss than the borax dosage (+28.68 vs. −13.55 in Equation (2)). As shown in Figure 4a, while keeping the borax dosage at the middle level (0.16), it is apparent that mini-slump loss decreased with decreasing M/P, whereas it decreased with increasing W/S. Moreover, the combined effects of W/S and borax dosage are shown in Figure 4b. As expected, the mini-slump loss decreased with increasing W/S and borax dosage. Similarly, as shown in Figure 4c, the mini-slump loss decreased with the decrease in M/P and increase in borax dosage. For example, when M/P was 7 and borax dosage was 0.16, the predicted mini-slump loss was 38.75. However, when M/P decreased to 4.03 while maintaining borax dosage at 0.16, the predicted mini-slump loss decreased to 10.23; meanwhile, it also decreased to 25.20 if increasing the borax dosage to 0.17 but keeping the M/P at 7.

#### 3.2.3. Effect on Yield Stress

**Figure 5:**
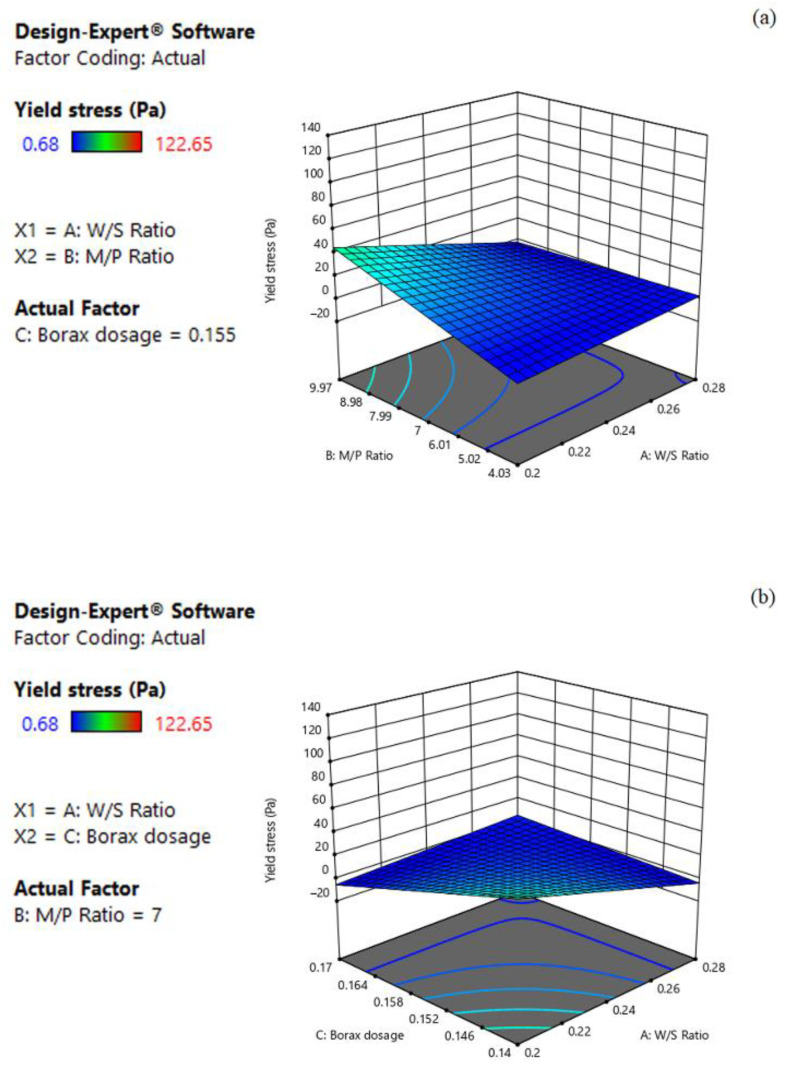

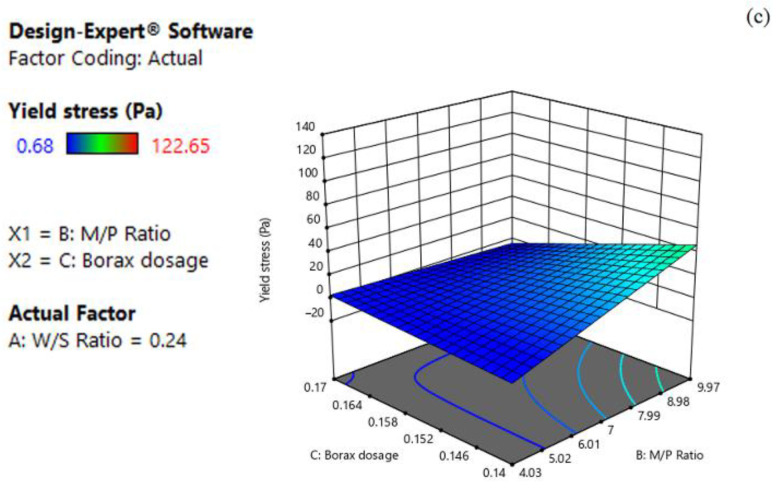
Three-dimension response surface plots of yield stress (Y_3_) in relation to: (**a**) W/S ratio (X_1_) and M/P ratio (X_2_); (**b**) W/S ratio (X_1_) and borax dosage (X_3_); (**c**) M/P ratio (X_2_) and borax dosage (X_3_).

The 3D response surface plots visualizing the effect of the three factors on the yield stress of MPC paste are plotted as the response surfaces in Figure 5. As shown in Equation (3), the yield stress response decreased with the increase in W/S and borax dosage, whereas it increased with the increase in M/P. Moreover, the influences in the order of decreasing magnitude are M/P ratio, borax dosage and W/S. For example, the coefficient of M/P showed an approximately 1.2 times greater effect on the yield stress than that of W/S (+11.60 vs. −9.67). As shown in Figure 5a, while keeping the borax dosage at the middle level (0.16), it is evident that the yield stress increased with increasing M/P, whereas it decreased with the increase in W/S. When W/S was 0.24 and M/P was 7, the response of the predicted yield stress value was 9.61 Pa. However, when W/S decreased to 0.20 while maintaining M/P at 7, the predicted yield stress value increased to 19.28 Pa, while it increased to 21.21 Pa if increasing M/P to 9.97 but keeping the W/S at 0.24. The combined effects of W/S and borax dosage are shown in Figure 5b. As expected, the yield stress decreased with increasing W/S and borax dosage. Similarly, as shown in Figure 5c, the yield stress increased with the increase in M/P, while it decreased with increasing borax dosage. There could be interactions between the three factors in relation to the yield stress. For example, the proportion of water could not only affect the fluidity, but also change the hydration process of the MPC [33]. Therefore, further studies still need to be carried out to clarify their roles.

#### 3.2.4. Effect on Plastic Viscosity

**Figure 6:**
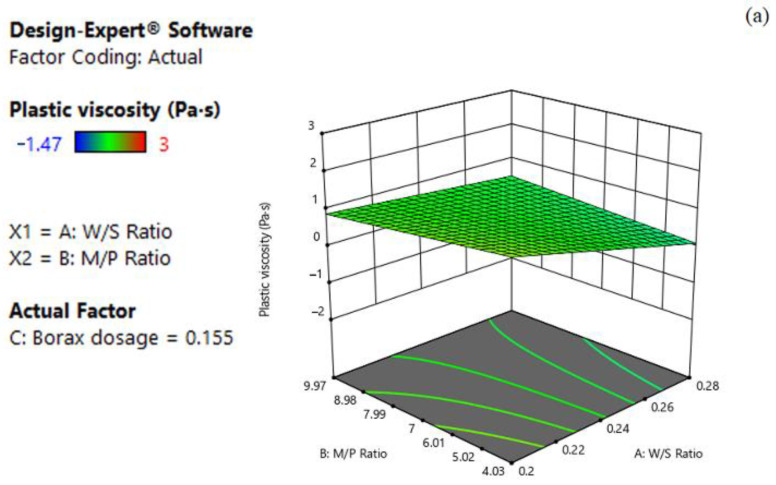

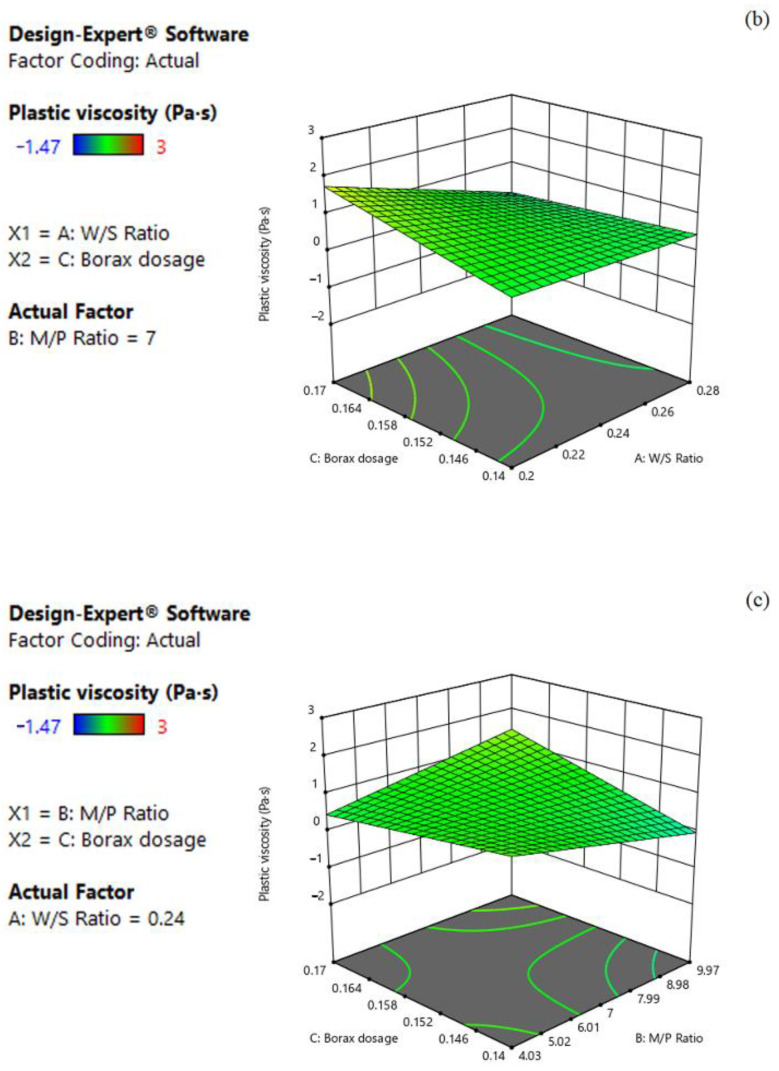
Three-dimension response surface plots of plastic viscosity (Y_4_) in relation to: (**a**) W/S ratio (X_1_) and M/P ratio (X_2_); (**b**) W/S ratio (X_1_) and borax dosage (X_3_); (**c**) M/P ratio (X_2_) and borax dosage (X_3_).

The 3D response surface plots based on Equation 4 are shown in Figure 6a–c. In general, the plastic viscosity response decreased with the increase in the W/S ratio and M/P ratio, while it increased with the increase in borax dosage. Moreover, it is obvious that the coefficient of the W/S (−0.43) was approximately 9.8 and 1.2 times higher than that of M/P and borax dosage, respectively, indicating that the W/S can significantly affect the plastic viscosity of MPC. The dependence of plastic viscosity on W/S and M/P for the set of borax dosage at its central level (0.16) is shown in Figure 6a. As can be seen, the plastic viscosity decreased with the increase in W/S, whereas the change in M/P imposed little effect on plastic viscosity. For example, the response of the predicted plastic viscosity was 0.73 Pa·s when the value of W/S and M/P were set as 0.24 and 7, respectively. However, when decreasing the W/S to 0.20 while maintaining the M/P at 7, the predicted plastic viscosity increased to 1.16 Pa·s. However, it only increased to 0.77 Pa·s if decreasing the M/P to 4.03 but keeping the W/S at 0.24. This phenomenon may be attributed to the fact that the change in W/S and MgO proportion could affect the cohesion of the fluids. Additionally, the effects of W/S and borax dosage on the plastic viscosity are presented in Figure 6b. The plastic viscosity decreased as the W/S increased towards its high level, while it increased along with increasing borax dosage. As illustrated in Figure 6c, the plastic viscosity increased with increasing borax dosage, while the change in M/P contributed little to plastic viscosity.

#### 3.2.5. Discussion

To compare the effects of each variable on the rheological behaviour of MPC, the coefficients of all regression models are summarised in Table 9. Since the p-value < 0.05 would indicate that the term is significant at 95% confidence, the significant terms for each response in Table 9 are bold.

As shown in Table 9, the W/S ratio mainly affected the initial mini slump and plastic viscosity. The increase in W/S led to the increase in mini slump, but the decrease in plastic viscosity. However, it should be noticed that, although yield stress was not the significant term at 95% confidence, it was still above the 90% confidence level, indicating that it still can be influenced by W/S. In a cementitious system, Van der Waals interactions have been recognised to dominate all colloidal interactions and, therefore, dictate the inter-particle distance [34]. The increased W/S reduces the concentration of the particles which, therefore, enlarges the inter-particle distance. Since the inter-particles force is inversely proportional to the square of inter-particle distance [35], the increased inter-particle distance due to the high W/S can reduce the inter-particle force. This, in turn, can reduce the formation of the solid network through particle–particle bonds [36]. Consequently, higher mini slump and lower yield stress can be achieved. Moreover, W/S is also identified as the most significant factor influencing the plastic viscosity of MPC. This could be attributed to the fact that the plastic viscosity can somehow reflect the flocculation status in the paste and the resistance to the flow, which in turn, depends on the solid volume fraction and the packed density [37]. The alteration of W/S changed the solid volume, consequently changing the plastic viscosity of MPC.

In terms of the M/P ratio, Table 9 demonstrates that it can significantly affect the mini-slump loss and yield stress. The higher M/P ratio shows a higher mini-slump loss and yield stress. It is well known that the mini-slump loss is closely related to the setting of MPC, whilst the yield stress is closely related to the hydration process. Although it is generally accepted that the main hydration product of MKPC is k-struvite (MgKPO_4_·6H_2_O), which is formed from the reaction between MgO and KH_2_PO_4_ [38] through solution, the different ratios between MgO and KH_2_PO_4_ can lead to a different reaction process and products [13]. Specifically, the increased portion of MgO could lead to a higher pH of MPC, which promotes the dissolution of the ions available for reaction [39], and more reaction products could form and precipitate on the particle surface [14]. Consequently, the hydration process could be accelerated and setting time shortened, resulting in a higher mini-slump loss. Furthermore, the produced negatively charged k-struvite or other intermedium products [40,41] from hydration may interact with the positively charged MgO particles [42], which could increase the yield stress by forming the electrostatic attraction force among the particles.

Similarly, borax dosage, another factor which is related to the hydration process, affected the mini-slump loss and yield stress. In contrast to the effect of M/P ratio, the increase in borax dosage reduced both mini-slump loss and yield stress. The addition of borax retarders delayed the setting of MPC [43]. As discussed previously, since the mini-slump loss is linked to the setting of MPC, the increase in the borax dosage reduced the mini-slump loss of MPC by delaying the setting of MPC. Moreover, the addition of borax retards the MPC hydration by formatting the positively charged MgB(OH)_4_^+^ complex [43]. Therefore, the electrostatic repulsion between the positively charged complex and MgO could benefit the dispersion of the particles, which can further reduce the yield stress of the MPC paste.

Furthermore, it should be noted that, based on statistical analysis the interaction between any two factors (X_1_X_2_, X_1_X_3_, X_2_X_3_) can still affect the yield stress of the MPC paste. Besides, the interaction between M/P and borax dosage (X_2_X_3_) exhibited a greater influence on mini-slump loss. Such interactions lead to a more complex situation in analysing the fresh property of MPC. Therefore, although the influence of each variable on the fresh property of MPC was discussed in detail, the interactions between these variables need to be further investigated.

### 3.3. Desirability Functions for Numerical Optimisation

Based on the results and the analysis presented in previous sections, the models for all four responses, in terms of initial mini slump, mini-slump loss, yield stress and plastic viscosity (Equations (1)–(4)), were utilised to optimise the best fresh property of MPC paste. Factors including W/S, M/P and borax dosage are involved in the numerical optimisation with the design goal as ‘in range’. On the other hand, the goals in terms of yield stress, plastic viscosity, initial mini slump and mini-slump loss are set as ‘minimised’, ‘in range’, ‘maximised’ and ‘minimised’ to obtain the MPC system with desirable fresh properties. Considering the ease to process the fresh MPC in its future engineering application, the importance of yield stress, initial mini slump and mini-slump loss was set as 5, whereas it was set as 3 (default value) for other terms. The factors and predicted response of the target are listed in Table 10. As shown in the table, the predicted values for the four responses generated from the optimisation are 0.680 Pa (yield stress), 0.263 Pa·s (plastic viscosity), 161.858 mm (initial mini slump) and 11.282 (mini-slump loss), with the highest desirability of 0.867. Accordingly, the optimum conditions of the three variables are 0.280, 7.528 and 0.170 for the W/S, M/P and borax dosage, respectively. Thus, it is anticipated that by following the optimum recipe computed for W/S, M/P and borax dosage, a MPC mix with desirable fresh properties can be obtained. Our paper reported for the first time the optimised rheological parameters of the MPC materials by the statistic RSM-CCD methodology.

### 3.4. Compressive Strength

To use MPC in practice, its compressive strength should be examined. Considering that MPC develops its strength very quickly at an early age, the 1 d and 7 d compressive strength of the paste cubes were tested, and the results are reported in Table 11. The strength values were not particularly high, which is due to the high W/S ratio used in this study, as reported in Table 1. For all mixes, the compressive strength increased with age (from 1 d to 7 d), which agrees with the results reported in the literature [17]. The development of the strength, which has been widely investigated, is due to the continuous generation of the hydration product i.e., struvite. It should be noted that the strength of the Run 15 is not available, which is due to the break-down of the cubes caused by expansion. It can be noticed that the compressive strength varied with the change of the three factors, i.e., W/S Ratio (X_1_), M/P Ratio (X_2_) and borax dosage (X_3_), indicating the potential effects of these factors on compressive strength. The relationships between the strength and the rheologic parameters, as well as the interactions between the variables and their combined effects on the strength and rheologic properties will be investigated in future.

## 4. Conclusions

Based on the results obtained, the following conclusions can be drawn:

- The RSM-CCD methodology was successfully adopted to investigate the rheological behaviour of MPC material and to optimise the mix proportion in terms of W/S, M/P and borax dosage, with initial mini slump, mini-slump loss, yield stress and plastic viscosity considered as responses.
- The W/S ratio was identified as the significant factor (95% confidence level) affecting the plastic viscosity and the initial mini slump. The increase in W/S led to the decrease in the plastic viscosity, whereas it increased the mini slump. Moreover, the influence on the yield stress could not be ignored, since it remained at a 90% confidence level.
- The yield stress and mini-slump loss were influenced by the M/P ratio. The increase in the M/P ratio was shown to increase the yield stress and mini-slump loss.
- Borax dosage clearly affected the yield stress and mini-slump loss of MPC. With the increase in borax dosage, the yield stress and mini-slump loss decreased.
- The numerical optimisation showed that the best predicted values for the four responses are 0.680 Pa (yield stress), 0.263 Pa·s (plastic viscosity), 161.858 mm (initial mini slump) and 11.282 (mini-slump loss), with the desirability of 0.867.

## Figures and Tables

**Table 1:** Actual values for the variables used in the experimental design.

Independent Variables	Symbols	Actual Values for the Coded Values				
		−α (−1.682)	−1	0	+1	+α (+1.682)
W/S ratio	X_1_	0.18	0.20	0.24	0.28	0.30
M/P ratio	X_2_	2	4.03	7	9.97	12
borax dosage	X_3_	0.13	0.14	0.16	0.17	0.18

**Table 2:** The points for 3 factors CCD design (coded value).

Runs	W/S Ratio (X_1_)	M/P Ratio (X_2_)	Borax Dosage (X_3_)
1	0	0	0
2	−1	1	−1
3	0	0	1.682
4	0	0	0
5	0	0	0
6	−1	−1	−1
7	−1.682	0	0
8	0	0	−1.682
9	1.682	0	0
10	1	1	−1
11	0	0	0
12	1	−1	1
13	−1	1	1
14	−1	−1	1
15	0	−1.682	0
16	0	0	0
17	1	1	1
18	0	0	0
19	1	−1	−1
20	0	1.682	0

**Table 3:** The points for 3 factors CCD design (coded value) and corresponding responses.

Runs	Variables in Coded Values			Responses			
	W/S Ratio (X_1_)	M/P Ratio (X_2_)	Borax Dosage (X_3_)	Initial Mini Slump/mm(Y_1_)	Mini-Slump Loss (Y_2_)	Yield Stress/Pa (Y_3_)	Plastic Viscosity/Pa·s (Y_4_)
1	0	0	0	159	33	2.03	0.77
2	−1	1	−1	140	102	122.65	−1.47
3	0	0	1.682	156	22	1.18	0.59
4	0	0	0	152	22	1.76	0.70
5	0	0	0	155	25	1.39	0.74
6	−1	−1	−1	142	12	3.57	1.42
7	−1.682	0	0	134	96	7.38	3.00
8	0	0	−1.682	156	22	10.96	0.81
9	1.682	0	0	164	6	1.17	0.36
10	1	1	−1	175	137	7.18	0.37
11	0	0	0	168	36	1.46	0.66
12	1	−1	1	155	10	0.68	0.33
13	−1	1	1	144	31	4.20	2.10
14	−1	−1	1	143	13	1.84	0.90
15	0	−1.682	0	156	23	0.98	0.31
16	0	0	0	161	31	1.13	0.69
17	1	1	1	158	20	1.85	0.46
18	0	0	0	147	17	1.40	0.72
19	1	−1	−1	160	8	0.90	0.31
20	0	1.682	0	147	109	18.50	0.88

**Table 4:** Repeatability of the responses at central points.

Test	Initial Mini Slump /mm	Mini-Slump Loss	Yield Stress /Pa	Plastic Viscosity /Pa·s
Mean (*n* = 6)	157.00	27.33	1.53	0.71
Standard Derivation	7.35	7.23	0.32	0.04
Standard Error	3.00	2.95	0.13	0.02
Coefficient of Variation (%)	4.68	26.45	20.78	5.44

**Table 5:** Analysis of variance (ANOVA) of the response model of initial mini-slump.

Source	Responses				
	Initial Mini-Slump (Y_1_)				
	Sum of Squares	DF	MS	F-Value	p-Value (Prob > F)
Model	1248.51	3	416.17	9.67	0.0007
X_1_	1227.10	1	1227.10	28.53	0.0001
X_2_	0.25	1	0.25	0.01	0.9397
X_3_	21.16	1	21.16	0.49	0.4931
Residual	688.29	16	43.02		
Lack of fit	418.29	11	38.03	0.70	0.7090
Pure Error	270.00	5	54.00		
Cor Total	1936.80	19			
R^2^	0.6446				
Adeq Precision	10.8699				

**Table 6:** Analysis of variance (ANOVA) of the response model of mini-slump loss.

Source	Responses				
	Mini-Slump Loss (Y_2_)				
	Sum of Squares	DF	MS	F-Value	p-Value (Prob > F)
Model	19,992.14	6	3332.02	5.18	0.0063
X_1_	1321.90	1	1321.90	2.06	0.1753
X_2_	11,230.80	1	11,230.80	17.46	0.0011
X_3_	2506.07	1	2506.07	3.90	0.0700
X_1_X_2_	120.13	1	120.13	0.19	0.6727
X_1_X_3_	253.13	1	253.13	0.39	0.5413
X_2_X_3_	4560.13	1	4560.13	7.09	0.0195
Residual	8361.61	13	643.20		
Lack of fit	8100.28	8	1012.54	19.37	0.0023
Pure Error	261.33	5	52.27		
Cor Total	28,353.75	19			
R^2^	0.7051				
Adeq Precision	7.6423				

**Table 7:** Analysis of variance (ANOVA) of the response model of yield stress.

Source	Responses				
	Yield Stress (Y_3_)				
	Sum of Squares	DF	MS	F-Value	p-Value (Prob > F)
Model	9716.05	6	1619.34	5.13	0.0066
X_1_	1277.66	1	1277.66	4.05	0.0655
X_2_	1836.17	1	1836.17	5.82	0.0314
X_3_	1480.18	1	1480.18	4.69	0.0496
X_1_X_2_	1624.22	1	1624.22	5.14	0.041
X_1_X_3_	1642.51	1	1642.51	5.2	0.0401
X_2_X_3_	1855.32	1	1855.32	5.88	0.0307
Residual	4104.84	13	315.76		
Lack of fit	4104.33	8	513.04	5086.84	<0.0001
Pure Error	0.5	5	0.1		
Cor Total	13,820.88	19			
R^2^	0.7030				
Adeq Precision	9.6694				

**Table 8:** Analysis of variance (ANOVA) of the response model of plastic viscosity.

Source	Responses				
	Plastic Viscosity (Y_4_)				
	Sum of Squares	DF	MS	F-Value	p-Value (Prob > F)
Model	6.84	6	1.14	2.3	0.0979
X_1_	2.57	1	2.57	5.18	0.0404
X_2_	0.02	1	0.02	0.04	0.8384
X_3_	0.57	1	0.57	1.15	0.3030
X_1_X_2_	0.44	1	0.44	0.89	0.3623
X_1_X_3_	1.08	1	1.08	2.18	0.1636
X_2_X_3_	2.16	1	2.16	4.37	0.0569
Residual	6.44	13	0.5		
Lack of fit	6.43	8	0.8	533.79	<0.0001
Pure Error	0.01	5	0.002		
Cor Total	13.28	19			
R^2^	0.5151				
Adeq Precision	6.1537				

**Table 9:** Coefficient table for the variables of all responses.

	Initial Mini Slump		Mini-Slump Loss		Yield Stress		Plastic Viscosity	
	Coefficient	p-Value	Coefficient	p-Value	Coefficient	p-Value	Coefficient	p-Value
Intercept	153.60		38.75		9.61		0.73	
X_1_	9.48	**<0.0001**	−9.84	0.1753	−9.67	0.0655	−0.43	**0.0404**
X_2_	0.14	0.9397	28.68	**0.0011**	11.60	**0.0314**	−0.04	0.8384
X_3_	−1.24	0.4931	−13.55	0.0700	−10.41	**0.0496**	0.20	0.3030
X_1_X_2_			3.88	0.6727	−14.25	**0.0410**	0.24	0.3623
X_1_X_3_			−5.63	0.5413	14.33	**0.0401**	−0.37	0.1636
X_2_X_3_			−23.88	**0.0195**	−15.23	**0.0307**	0.52	0.0569

Note: X_1_: W/S Ratio; X_2_: M/P Ratio; X_3_: borax dosage; X_1_X_2_: interaction between W/S ratio and M/P ratio; X_1_X_3_: interaction between W/S ratio and borax dosage; X_2_X_3_: interaction between M/P ratio and borax dosage.

**Table 10:** Characteristics of numerical optimisation.

Parameters	Importance	Weight	Goal	Predict Value
W/S Ratio (X_1_)	3	1	In range	0.280
M/P Ratio (X_2_)	3	1	In range	7.528
Borax Dosage (X_3_)	3	1	In range	0.170
Yield Stress/Pa	5	1	Minimise	**0.680**
Plastic Viscosity/Pa·s	3	1	In range	**0.263**
Initial Mini Slump/mm	5	1	Maximise	**161.858**
Mini-Slump Loss	5	1	Minimise	**11.282**
Desirability	**0.867**			

**Table 11:** The compressive strength of the cube specimens.

Runs	1 d Compressive Strength/MPa	7 d Compressive Strength/MPa
1	23.7	34.6
2	33.8	34.4
3	19.1	39.0
4	21.8	32.2
5	23.3	33.2
6	22.4	33.8
7	27.5	43.1
8	29.2	32.3
9	13.7	15.8
10	9.2	10.6
11	21.0	30.8
12	18.5	22.8
13	21.7	22.9
14	21.5	31.9
15	n.a.	n.a.
16	21.7	32.8
17	11.1	11.6
18	19.9	27.2
19	10.2	14.1
20	7.2	8.2

## Data Availability

Data are contained within the article.
